# Pediatric Cochlear Implants in the Chudley-McCullough Syndrome: A Report of Two Cases

**DOI:** 10.7759/cureus.55440

**Published:** 2024-03-03

**Authors:** Noah E Alter, Kailey Introcaso, Cathy Nunez, Leonor Roach, Samuel T Ostrower

**Affiliations:** 1 Department of Medical Education, Dr. Kiran C. Patel College of Allopathic Medicine, Nova Southeastern University, Davie, USA; 2 Department of Audiology, Joe DiMaggio Children’s Hospital, Hollywood, USA; 3 Department of Speech-Language Pathology, Joe DiMaggio Children’s Hospital, Hollywood, USA; 4 Department of Pediatric Otolaryngology, Joe Dimaggio Children’s Hospital, Hollywood, USA

**Keywords:** cochlear implant, inner ear function, pediatric audiology, cochlear implant (ci) surgery, sensorineural deafness, chudley-mccullough syndrome

## Abstract

Chudley-McCullough syndrome (CMS) is a rare autosomal recessive disorder characterized by sensorineural hearing loss and cerebral abnormalities, including ventriculomegaly and partial dysgenesis of the corpus callosum. CMS is caused by two inactivating mutations of the G protein signaling modulator 2 (GPSM2), which maintains inner hair cell polarity and spindle orientation. Since its initial description, CMS has been reported approximately 30 times in the medical literature with several individuals undergoing cochlear implantation to restore their hearing. Interestingly, within the past two years, we encountered two cases of CMS in our hospital, which primarily serves patients within a 30-mile radius. To our knowledge, the literature has yet to evaluate two unrelated cases of CMS occurring in such close succession. This case report describes two successful cases of bilateral cochlear implantation in two children with CMS. Notably, these individuals have no family history of consanguinity or prior hearing loss.

## Introduction

Chudley-McCullough syndrome (CMS) is an exceptionally rare autosomal recessive genetic disorder, primarily arising from mutations within the G protein signaling modulator 2 (GPSM2) gene. First delineated in medical literature in 1997, CMS manifests with a distinctive clinical phenotype marked by bilateral sensorineural hearing loss and cerebral abnormalities, notably hypoplasia of the corpus callosum [1,2]. The interplay between these cardinal features underscores the complex pathogenesis of CMS.

At the molecular level, CMS is intricately tied to disruptions in actin polymerization, particularly during the elongation of stereocilia within auditory and vestibular hair cells [3,4]. Mutations in GPSM2 disrupt this regulatory process, leading to aberrant stereocilia morphology and subsequent dysfunction of inner ear hair cells, culminating in profound sensorineural hearing loss [5].

Since its initial description, cochlear implantation stands as a promising treatment option for individuals with CMS afflicted by profound sensorineural hearing loss [6]. Despite the rarity of CMS, the debilitating nature of its auditory manifestations necessitates explorations of interventions to ameliorate communication challenges and improve quality of life. Of the seven reported cases of cochlear implantation in CMS individuals, all have demonstrated significant improvements in auditory function, with some achieving near-normal hearing thresholds postoperatively [3-6]. However, the limited number of reported cases underscores the need for further investigation into the long-term effectiveness and potential complications of cochlear implantation in this population.

Moreover, successful cochlear implantation in CMS individuals necessitates a comprehensive, multidisciplinary approach encompassing preoperative assessment, surgical intervention, and postoperative rehabilitation [7]. Close collaboration between otolaryngologists, audiologists, geneticists, and rehabilitation specialists is paramount to optimize patient outcomes and address the unique challenges posed by CMS. In this case report, we outline two successful cases of bilateral cochlear implantation in two young children with CMS diagnosed shortly after birth.

## Case presentation

Case 1

Patient 1, a two-year-old child, was referred to our pediatric cochlear implant center as an infant following abnormal diagnostic auditory brainstem response (ABR). The patient had no family history of deafness and was born via cesarean section after a full-term pregnancy notable for abnormal fetal imaging suggestive of hydrocephalus. Apgar scores were 8/1 and 9/5, birth weight was 2806 g, and head circumference measured 35 cm. Notably, patient 1 presented with dysmorphic features, including a large forehead and long philtrum.

**Figure 1:**
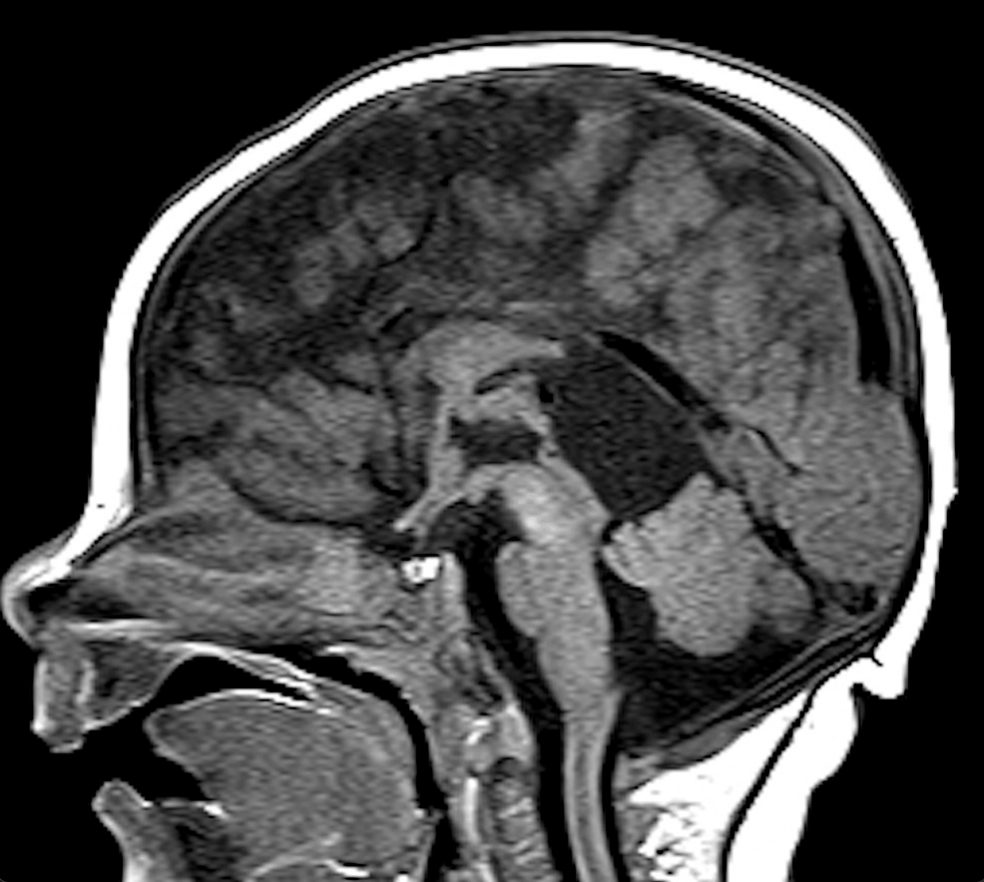
MRI of patient 1's brain (axial view). Colpocephaly associated with hydrocephalus of the temporal & atria-occipital horns and posterior bodies of the bilateral lateral ventricles.

Ultrasound of the patient's head on the day of life (DOL) 0 revealed bilateral symmetric dilation of the lateral ventricles, particularly pronounced in the occipital horns. However, subsequent MRI findings were suggestive of colpocephaly rather than hydrocephalus (Figure 1).

Audiology was consulted, and a comprehensive hearing evaluation, including ABR and otoacoustic emissions (OAE), was conducted. The results showed the presence of bilateral distortion product otoacoustic emissions (DPOAE) at all frequencies, accompanied by near-perfect reversals in polarity, indicating the presence of auditory neuropathy spectrum disorder (ANSD).

At eight months of age, patient 1 was fitted with binaural hearing aids. However, aided testing at 10 months revealed continued hearing loss within the moderately severe to severe range, and a speech evaluation demonstrated limited verbal skills, with no progress in receptive and expressive language. Genetic testing confirmed the diagnosis of CMS, identifying two biallelic pathogenic variants of the GPSM2 gene.

Further imaging, including an MRI of the internal auditory canals, labyrinth, and cranial nerves bilaterally, showed normal findings. At 15 months of age, patient 1 underwent successful bilateral cochlear implantation using a round window approach. The surgery was uneventful, with no cerebrospinal fluid gushers observed, and neural response imaging (NRI) results were within normal limits.

Eleven months after the activation of the cochlear implants, the patient's speech has shown significant improvement. They are now able to use one- to two-word phrases and follow commands with moderate prompts/cues.

Case 2

Patient 2, a two-year-old child, unrelated to patient 1, presented to our pediatric cochlear implant center at the age of one month following abnormal ABR. The patient's mother had a medical history of varicella and human papillomavirus, which were asymptomatic at the time of birth. The patient was born prematurely via cesarean section at 34 weeks of gestation. Apgar scores were 8/1 and 9/5, birth weight was 3080 g, and head circumference measured 33 cm. Patient 2 was born without craniofacial abnormalities; however, fetal ultrasound in the third trimester identified an intracranial cyst.

**Figure 2:**
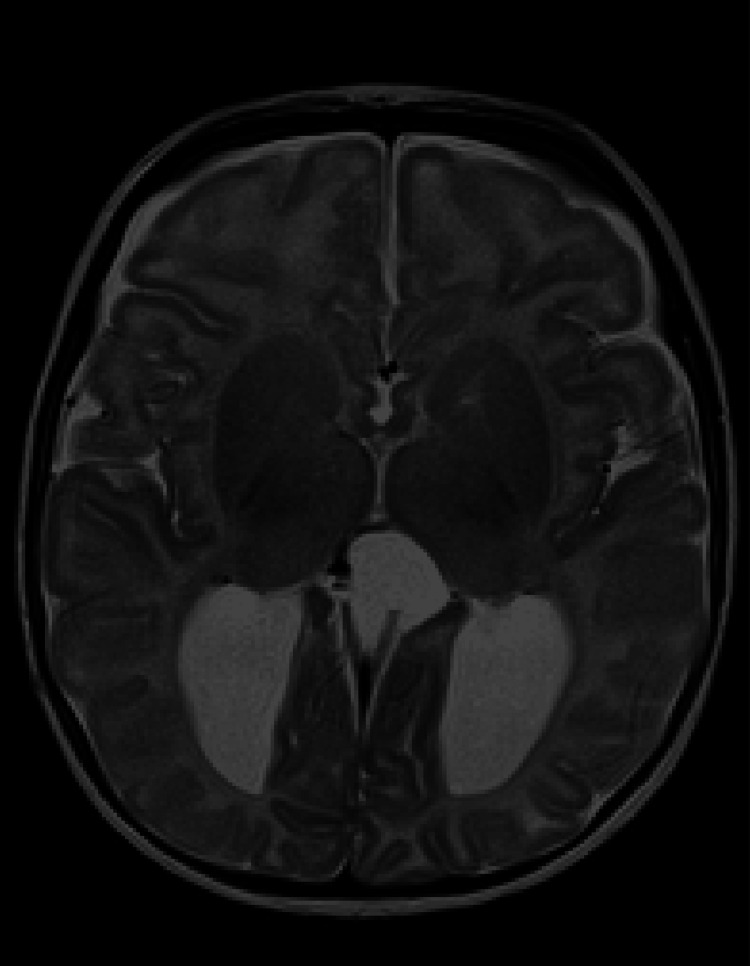
MRI of patient 2's brain (axial view). Colpocephaly with dysgenesis of the corpus callosum and third ventricle dilation and projection superiorly as a left paracentral dorsal cyst.

Subsequent head ultrasound after birth showed dilation of the lateral ventricles, particularly in the posterior horns, consistent with colpocephaly. MRI of the brain confirmed the presence of a midline interhemispheric pineal cyst, colpocephaly, and dysgenesis of the posterior corpus callosum (Figure 2).

Audiology was consulted, and a comprehensive hearing evaluation, including ABR and OAE, was conducted. The results showed present bilateral DPOAE at all frequencies, but with absent neuronal response and cochlear microphonic, indicating the presence of ANSD.

At three months of age, patient 2 was fitted with binaural hearing aids. Subsequent genetic evaluation identified two pathogenic variants of the GPSM2 gene, confirming the diagnosis of CMS. Despite the hearing aids, persistent delays in language development were observed, prompting an MRI of both ears. An MRI of the internal auditory canals, labyrinth, and cranial nerves revealed normal findings bilaterally. At 12 months of age, the patient underwent successful bilateral cochlear implantation using a round window approach. The procedure was uncomplicated and the NRI results were within the normal range for both ears.

Ten months after the activation of the cochlear implants, patient 2 has shown significant improvement in hearing and speech abilities. The patient is now able to use one-word phrases and imitate "learning to listen sounds.”

## Discussion

CMS is a rare autosomal recessive disorder characterized by sensorineural hearing loss and cerebral abnormalities, including ventriculomegaly and partial dysgenesis of the corpus callosum. Since its initial description in 1997, CMS has been reported approximately 30 times in the medical literature with several individuals undergoing cochlear implantation to restore their hearing. CMS is caused by two inactivating mutations of the GPSM2. GPSM2 is responsible for the maintenance of inner hair cell polarity and spindle orientation, which is critical for the development of early hearing [1]. In this report, we detail two individuals who were diagnosed with CMS and underwent cochlear implantation for auditory neuropathy spectrum disorder. Notably, these individuals have no history of consanguinity or prior hearing loss.

The prevalence of CMS in the United States is quite rare, affecting fewer than one in 1,000,000 individuals and fewer than 1000 people overall [2,3]. Interestingly, within the past two years, we encountered two cases of CMS in our cochlear implant center, which primarily serves patients within a 30-mile radius. It is worth noting that a majority of CMS patients present with significant structural brain abnormalities, but they do not typically experience cognitive impairment [2,3].

In examining the radiographic and clinical findings of CMS in case 1 and case 2, notable similarities and differences emerge. Both cases exhibited characteristic features such as colpocephaly, dysgenesis of the corpus callosum, and intracranial abnormalities, consistent with the known spectrum of CMS manifestations. However, case 1 presented with additional dysmorphic features, including a large forehead and long philtrum, whereas case 2 displayed an intracranial cyst in the third trimester of pregnancy. These variations underscore the heterogeneity of CMS phenotypes and emphasize the importance of individualized clinical assessment and management. Regarding consanguinity, while CMS is known to predominantly occur in populations with high rates of consanguineous marriages, such as certain Middle Eastern and North African communities, the absence of consanguinity in our two cases challenges this notion [3,5]. This observation suggests that genetic factors beyond consanguinity may contribute to the prevalence of CMS, warranting further investigation into the underlying genetic mechanisms and environmental influences driving the syndrome's occurrence.

These findings align with our two patients and are consistent with a previous report by Forli et al., who described a 31-year-old CMS patient with profound bilateral sensorineural hearing loss and colpocephaly [4]. Remarkably, the patient developed good oral language skills through intensive aural rehabilitation and exhibited normal cognitive abilities from birth. Similarly, both of our patients exhibited normal cognitive development and were born with colpocephaly.

In line with the management of most types of hearing loss, the initial treatment approach for our patients involved amplification with hearing aids [5,6]. However, similar to other CMS patients, the hearing aids yielded unsatisfactory outcomes, and subsequent testing revealed impaired receptive and expressive language abilities. Following the unsuccessful trial of amplification, our patients were deemed suitable candidates for cochlear implantation. To ensure normal inner ear anatomy, an MRI was performed, which confirmed the integrity of the internal auditory canals, labyrinth, and cranial nerves. This is consistent with the literature, which has shown no reported anomalies in the facial nerve, cochlea, auditory nerve, and petrous bone of CMS patients [1,3,4]. Following a comprehensive evaluation, both patients underwent successful bilateral cochlear implantation without intraoperative complications. Of note, prior reports of CMS mention sensorineural hearing loss, yet both of our patients were found to have auditory neuropathy spectrum disorder.

The postoperative outcomes of these cases have been favorable. Less than a year after cochlear implant activation, significant improvements in speech have been observed in both patients. Patient 1 has progressed to using one- to two-word phrases with moderate prompts/cues, while patient 2 has shown notable enhancements in hearing and speech abilities, demonstrating one-word phrases and imitating "learning to listen sounds." These results underscore the importance of early detection and intervention in CMS patients.

## Conclusions

This case report describes two successful cases of bilateral cochlear implantation in pediatric patients diagnosed with CMS. These cases provide evidence supporting the notion that early detection and intervention in pediatric patients with CMS can have positive long-term outcomes on speech and language development. Furthermore, these cases validate the safety and effectiveness of cochlear implantation for pediatric patients with CMS.
